# The Effect of Recently Developed Synbiotic Preparations on Dominant Fecal Microbiota and Organic Acids Concentrations in Feces of Piglets from Nursing to Fattening

**DOI:** 10.3390/ani10111999

**Published:** 2020-10-30

**Authors:** Agnieszka Chlebicz-Wójcik, Katarzyna Śliżewska

**Affiliations:** Institute of Fermentation Technology and Microbiology, Faculty of Biotechnology and Food Sciences, Lodz University of Technology, 90-924 Łódź, Poland

**Keywords:** synbiotics, microbiota, pigs

## Abstract

**Simple Summary:**

Widespread antibiotic resistance among microorganisms led to a prohibition or limitation of using antibiotic growth promoters in livestock breeding. In order to maintain the animal production on the level which could satisfy the demands, and to reduce the risk of infections occurrence among the livestock, alternative preparations are being searched for. Pro- and prebiotics are wildly studied; however, their combination, which are called synbiotics, are expected to impact animals’ health more considerably. There are a number of pro- and prebiotic preparations available on the market; nevertheless, synbiotics are rare, which is why this research was focused on their impact on pigs’ intestinal microbiota and organic acids synthesis. The results showed that newly developed synbiotics could have a more beneficial impact on piglets’ health rather than commercial probiotics.

**Abstract:**

The study was conducted to determine the influence of newly elaborated synbiotic preparations on piglets’ intestinal microbiota and its metabolism. Animals were distributed among six experimental groups, in reference to used feed supplements, namely, synbiotics (A, B, or C) or commercially available probiotics (BioPlus 2B^®^, Chr. Hansen A/S, Horsholm, Denmark or Cylactin^®^ LBC, DSM Nutritional Products Ltd., Kaiseraugst, Switzerland), or its absence (control group). Until the 29th day of life, piglets were breastfed by sows, whose feed was supplemented, and fecal samples were collected at the 7th and 28th day of piglets’ life. After weaning of the piglets, the research was continued until the 165th day of the pigs’ life. The area of this work included the analysis of the piglets’ dominant fecal microbiota by the plate count method. Moreover, high-performance liquid chromatography analysis (HPLC) was applied to establish variations in the concentrations of organic acids, namely, lactic acid, short-chain fatty acids (SCFAs), and branched-chain fatty acids (BCFAs). It was observed that synbiotics have a more significant beneficial effect on the intestinal microbiota of piglets and their metabolism, and therefore their health, in comparison to commercial probiotics used individually. Moreover, synbiotic preparations prevent the negative impact of weaning on piglets’ microbial population in the gastrointestinal tract, which could reduce the occurrence of diarrhea.

## 1. Introduction

The mammalian gastrointestinal tract (GIT) is colonized immediately after birth, by a heterogeneous and diversified ecosystem comprised of microorganisms. This microbial population, along with synthesized microbial products, pose a barrier against pathogens and support various vital morphological, immunological, and digestion functions as well as modulate the host’s genes expression [1].

Inhabitation of GIT is a dynamic process, which is affected by the mode of delivery, surrounding environment, gestational age, and genetics [2]. Sows’ fecal and vaginal microbiota are also a significant source of initial GIT microbiota for their offspring [3]. Before the second day of a piglets’ life, their GIT is colonized mainly by bacteria belonging to the *Escherichia, Clostridium, Fusobacterium, Streptococcus,* and *Enterococcus* genus. In the following days, more species, such as *Lactobacillus, Bacteroides, Prevotella,* and *Ruminococcus*, inhabit piglets’ GIT [4]. Furthermore, sows’ colostrum and breast milk can influence the development of GIT microbiota, immune system development, and gut maturation of piglets, which is associated with a prevalence of bioactive molecules, such as antibacterial peptides, growth factors, and oligosaccharides [5,6].

Weaning is a stressful time for piglets, as a result of several ongoing diets and environmental and social challenges that can have an impact on the immune system and GIT imbalance, including rapid changes in the microbiota [7,8]. In current swine production systems, weaning is practiced at the third–fourth week of piglets’ life and can affect the absorptive capacity of the small intestine, which can lead to fasting or even weaning anorexia, consequently, reducing the growth rate and feed efficiency [9,10]. During the weaning period, their feed is transitioned from liquid, high-fat, low-carbohydrate milk into dry, high-carbohydrate, and low-fat fodder. Moreover, piglets are separated from their mother, transported into new housing, and mixed with other animals in a herd [8,11]. The activation of inflammatory response pathways, gastric motility reduction, increased permeability to antigens and toxins, disruption of the mucin layer, as well as unfavorable changes in microbiota can be observed in the GIT of piglets that have been weaned from their mothers [10,12]. These disorders can not only lead to economic losses but also a higher prevalence of pathogenic bacteria in swine, and therefore, in porcine meat, which poses a threat to public health [13].

Before the European Union prohibited the usage of antibiotic growth promoters (AGPs), they were widely used to prevent disorders associated with weaning and to promote piglets’ growth [14]. However, the development of antibiotic-resistant pathogens and the occurrence of antibiotics residue in meat led to the implementation of management strategies, which require the use of substances alternative to AGPs [15]. To restore balance in GIT microbiota and control the spread of pathogens, nonantibiotic feed supplements are used in the swine industry, such as zinc oxide, essential oils, organic acids, pro-, pre-, and synbiotics, as well as antimicrobial peptides and bacteriophages [10,16].

Live microorganisms, such as bacteria from genera *Bifidobacterium* or *Lactobacillus,* are used as feed additives, which when administered confer a health benefit on the host are called probiotics [17,18]. Prebiotics, on the other hand, are substrates that have a positive impact on the host as a consequence of being selectively metabolized by microorganisms [19]. Synbiotics, by combining pro- and prebiotics can enhance the positive effects of these components [20].

The described research aimed to determine the influence of newly developed synbiotic preparations on dominant microorganisms of GIT microbiota of suckling piglets and then in weaners and finisher pigs. Moreover, the lactic acid, short-chain fatty acids (SCFAs), and branched-chain fatty acids (BCFAs) concentrations were determined in piglets’ feces when the feed additives were used.

## 2. Materials and Methods

### 2.1. Feed Additives

The impact of newly developed synbiotic preparations on intestinal microbiota of piglets was analyzed. Commercial probiotics, namely, BioPlus 2B^®^ (Chr. Hansen A/S, Horsholm, Denmark) and Cylactin^®^ LBC (DSM Nutritional Products Ltd., Kaiseraugst, Switzerland) were used as the reference preparations. All synbiotics were composed of probiotics, namely, 10^7^ CFU/g *Saccharomyces cerevisiae* ŁOCK 0119 and 10^9^ CFU/g *Lactobacillus* spp. strains, as well as 20 g/kg of inulin as a prebiotic (Table 1).

Probiotic strains, after being isolated by the Institute of Fermentation Technology and Microbiology (IFTM; Lodz University of Technology, Poland) within the PBS/A8/32/2015 project, were deposited in the Lodz Collection of Pure Cultures 105 (ŁOCK 105). Probiotic *Lactobacillus* spp. strains and *S. cerevisiae* ŁOCK 0119 included in synbiotics were described in previous works and the patent applications [21,22,23,24,25,26,27,28,29].

### 2.2. Animals Treatment

Production of Danbred sows and their piglets was conducted at the “all-in, all-out” method at a farm in Poland owned by a private breeder. The National Veterinary Research Institute (Puławy, Poland) oversaw the rearing of the herd, which was housed indoors in high-standard facilities, as well as conducting a serological, pathological, and clinical analysis to control the animals’ health. Pseudorabies virus (PRV), porcine reproductive and respiratory syndrome virus (PRRSV), swine influenza virus (SIV), and *Mycoplasma hyopneumoniae* (Mhp) were not detected. Moreover, animals were free from pathogens common for the respiratory system, such as *Streptococcus suis*, *Actinobacillus pleuropneumoniae,* and *Haemophilus parasuis*, or the GIT, namely, *Clostridium perfringens, Brachyspira hyodysenteriae,* and verotoxic *E. coli* [30]. 

The basis for sows (*n* = 30) selection from the herd was their weight (256.7 ± 16.4 kg). The animals were distributed between six treatment groups five sows each, according to used supplement (synbiotics or probiotics), for which administration with feed (Table 2) begun 10 days before farrowing and was continued for 48 days, including the period of lactation [30,31].

Piglets were chosen randomly from each litter, ear-tagged, and divided into six groups (*n* = 8 each) depending on the feed additive given to their mother or its absence (Table 1). Piglets assigned to groups A, B, and C were breastfed by sows fed fodder with the addition of synbiotics A, B, and C accordingly, and after being weaned off (29th day of life), piglets were fed directly with feed supplemented with the corresponding synbiotic preparation. For piglets that were breastfed by sows assigned to groups D and E, who were fed fodder with one of the commercial probiotics, the feed was also supplemented directly with BioPlus 2B^®^ (Chr. Hansen A/S, Horsholm, Denmark; group D), or Cylactin^®^ LBC (DSM Nutritional Products Ltd., Kaiseraugst, Switzerland; group E) after weaning. All preparations were administrated in the amount of 0.5 kg per 1 ton of fodder (Table 2). Animals assigned to the control group (K), both sows and piglets after weaning, were administrated appropriate feed (Table 2) without any supplements.

**Figure 1:**
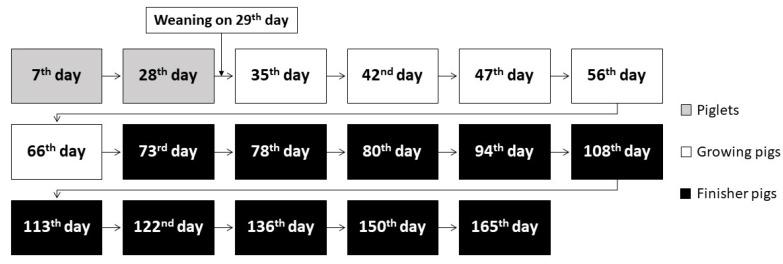
Feces sample collection across piglets’ maturation, during the conducted research. To avoid data overload, only the results obtained from samples collected at the beginning and the end of each growth phase were presented in the following paper.

First samples of feces were gathered 7 days after the piglets’ birth and this continued until animals reached the age of 165 days (Figure 1). The National Veterinary Research Institute was responsible for collecting samples that were then transferred to the IFTM and subjected to proper assays.

The 2nd Local Ethics Review Committee for Animal Experiments (Lublin, Poland) approved the following study on 22 January 2015 (resolution no. 4/2015).

### 2.3. Methods

#### 2.3.1. Fecal Dominant Microorganisms Population Determination

Samples were prepared by suspending collected feces (1 g) in sterile saline solution (9 mL); subsequently, they were diluted decimally. A cultivation-based analysis (the plate count method) with selective microbiological agar media was carried out to verify the structure of dominant fecal microbiota. The total number of anaerobic bacteria was established with Plate Count Agar (PCA; Merck Millipore, Darmstadt, Germany), whereas for *Bifidobacterium* sp., *Lactobacillus* sp., and *Bacteroides* sp., Reinforced Clostridial Agar (RCA; Oxoid™, Thermo Fisher Scientific, Waltham, MA, USA), de Man, Rogosa, and Sharpe (MRS; Merck Millipore, Darmstadt, Germany) agar, and VL (Oxoid ™, Thermo Fisher Scientific, Waltham, MA, USA) agar were used, respectively. Furthermore, the population density of *Clostridium* sp., *Enterococcus* sp., and yeast was enumerated with the usage of Tryptose Sulphite Cycloserine (TSC; Merck Millipore, Darmstadt, Germany), Bile Aesculin Agar (BAA; Merck Millipore, Darmstadt, Germany), and Sabouraud Dextrose Agar (SDA; Merck Millipore, Darmstadt, Germany), accordingly. The number of bacteria belonging to *Enterobacteriaceae* family was determined with McConkey Agar (Merck Millipore, Darmstadt, Germany), whereas for the selective cultivation of *Escherichia coli* Tryptone Bile X-glucuronide (TBX; Merck Millipore, Darmstadt, Germany) was used. Cultivations were conducted in accordance with appropriate Polish Norms (Table 3).

After the incubation process, colonies on the Petri dishes were counted and the results were given as the decimal logarithm of colony-forming units per 1 g of a sample (log_10_CFU/g). Every sample was tested in three replications.

#### 2.3.2. Organic Acid Concentrations Analysis

Suspensions of 0.5 g of feces were made in 1 mL of sterile demineralized water by vortexing for 3 min. Afterward, samples were centrifuged (Gusto^®^ High-Speed Mini Centrifuge; Heathrow Scientific LLC, Vernon Hills, IL, USA) for 8 min at 10,000 rpm. With the usage of 0.22 μm polytetrafluoroethylene (PTFE) syringe filters (Millex-GS, Merck Millipore, Darmstadt, Germany), the supernatants were filtered and subjected to high-performance liquid chromatography analysis (HPLC). 

Organic acids, namely, lactate, SCFAs (acetate, propionate, butyrate, valerate), and BCFAs (isobutyrate, isovalerate) concentrations were established with the usage of the Surveyor HPLC System (Thermo Scientific, Waltham, MA, USA) equipped with the Aminex HPX-87H column of 300 × 7.8 mm in dimensions (Bio-Rad Laboratories, Hercules, CA, USA). The parameters of the analysis are presented in Table 4.

Retention times of HPLC standards were used to detect analyzed organic acids on chromatograms. Moreover, the concentration of acids was calculated with the use of the area under the established peak based on previously prepared standard curves. The results were given as micromole per gram (µmol/g).

### 2.4. Statistical Analysis

From each experimental group of animals, six samples were tested in three replications; therefore, the presented results are arithmetic mean values.

Obtained outcomes were presented with heatmaps which were created with the ClustVis web tool (https://biit.cs.ut.ee/clustvis/). Input data of microbial counts in feces of piglets fed fodder with tested additives or its absence was given as log_10_CFU/g and they were not scaled or transformed. However, since the results of organic acids concentrations (µmol/g) in fecal samples had the multiplicative relation, the ln(x) function was used to transform the results. Additionally, the results were clustered based on Euclidean distance and average linkage to present dendrograms of hierarchical clustering analysis (HCA). Furthermore, Principal Component Analysis (PCA) was performed to visualize correlations between used feed supplementations and dominant microbial population in GIT as well as organic acids production with the usage of XLSTAT Software (Addinsoft, SARL, Paris, France).

## 3. Results

Śliżewska and Chlebicz reported previously the influence of analyzed synbiotics on lactating sows’ fecal microbiota and lactate, SCFAs, and BCFAs concentrations [31]. These animals breastfed piglets that were studied in the following research. Moreover, the National Veterinary Research Institute performed the analysis of piglets’ growth performance, and only this research team is the authorized party for these detailed outcomes publication. Nevertheless, it was observed that synbiotic A did not have any influence on the growth performance of piglets; however, synbiotics B and C had a more beneficial impact than Cylactin^®^ LBC (DSM Nutritional Products Ltd., Kaiseraugst, Switzerland) but less substantial than BioPlus 2B^®^ (Chr. Hansen A/S, Horsholm, Denmark).

### 3.1. Faecal Microbiota

**Figure 2:**
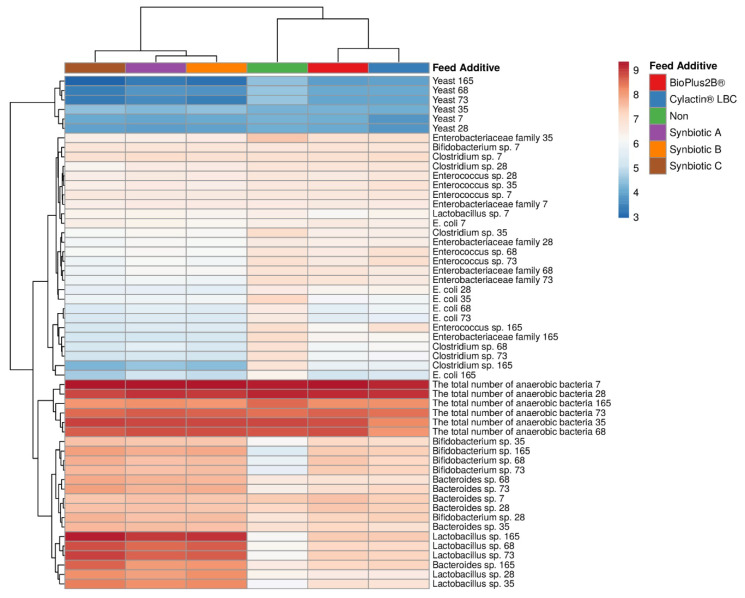
Heatmap presents changes in piglets’ fecal microbiota in correlation with the administrated feed additives. Columns correspond to the preparations used to modify piglets feed (directly or indirectly), whereas rows are assigned to specific microorganisms and the day of piglets’ life when fecal samples were collected, namely, 7th and 28th (suckling piglets), 35th and 68th (weaned piglets), and 73rd and 165th (finisher pigs).

It was observed that both newly developed synbiotics and probiotics (BioPlus 2B^®^, Cylactin^®^ LBC) contributed to a significant decrease in the number of potential pathogens, as well as an increase in beneficial bacteria population, such as *Lactobacillus* sp. and *Bifidobacterium* sp. in pigs’ feces. Nevertheless, the influence of synbiotics was more substantial than the influence of probiotics, which is demonstrated by vertical dendrograms (Figure 2). They indicate that the impact of both commercial probiotic preparation on pigs’ fecal microbiota was closer to the one caused by unmodified feed rather than the effect of synbiotics, especially preparation C.

**Figure 3:**
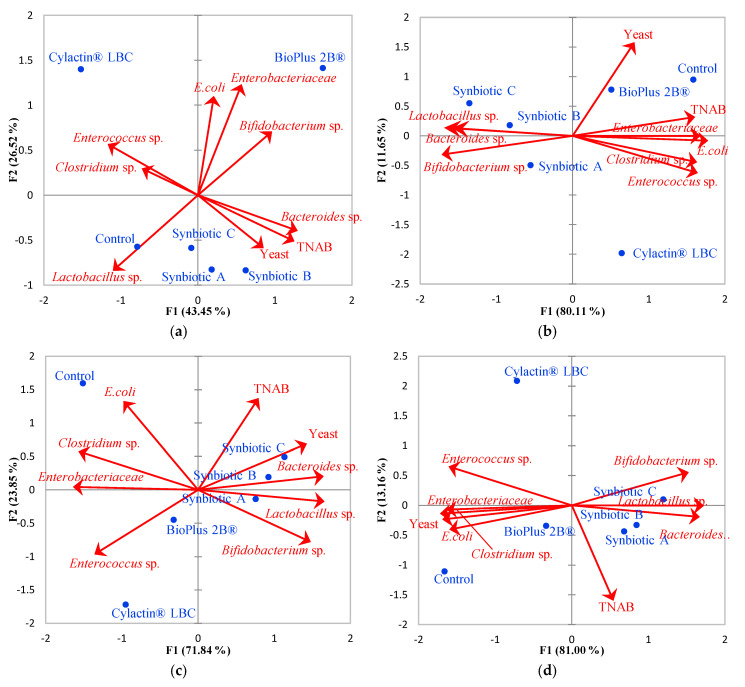

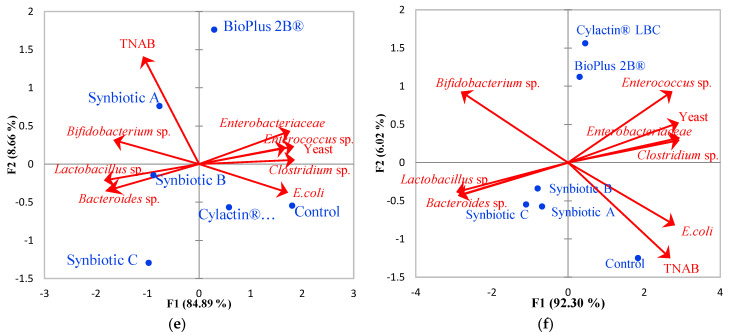
Correlation biplots which display the PCA results of the prevalent fecal microbiota (red vectors which represent variables) of pigs at a different age, which were administrated unmodified feed or with different additives (blue dots) and their impact on the microbial community (TNAB stands for the total number of anaerobic bacteria). Biplots were prepared for each sampling day: (**a**) 7th and (**b**) 28th day of life—piglets breastfed by sows; (**c**) 35th, (**d**) 68th, (**e**) 73rd, and (**f**) 165th day of life—feed additives administrated directly to weaned piglets.

Principal component analysis (PCA) was performed, and based on its results (Figure 3), it was noted that positive relations between synbiotics used as feed additives and beneficial bacteria counts, such as *Lactobacillus* sp. and *Bifidobacterium* sp., were becoming more significant throughout the study. Administration of synbiotics to breastfeeding sows, and then directly to weaned piglets, resulted in a considerable increase of the number of *Lactobacillus* sp. from 6.31–6.40 log_10_CFU/g (average of 6.37 log_10_CFU/g) to 8.97–9.26 log_10_CFU/g (mean 9.09 log_10_CFU/g) in piglets’ feces (Table S1). Analogous dependency was observed in the synbiotics impact on *Bifidobacterium* sp. number, which was increased by almost one decimal logarithmic unit, to the level of 7.78–7.96 log_10_CFU/g (mean 7.86 log_10_CFU/g) in feces of 165-day old finisher pigs (Table S1). Feed supplementation with probiotics substantially elevated the abundance of *Lactobacillus* sp. and *Bifidobacterium* sp. in piglets’ feces; nevertheless, only to the average level of 7.41 log_10_CFU/g and 7.36 log_10_CFU/g, respectively, in piglets 165th day of life (Table S1). Synbiotics administration also led to the considerable increase in the number of *Bacteroides* sp. in piglets’ feces, from the average of 7.47 log_10_CFU/g to 8.25 log_10_CFU/g, whereas supplementation of BioPlus 2B^®^ (Chr. Hansen A/S, Horsholm, Denmark), as well as feeding piglets unmodified fodder, resulted in a significant decrease in the *Bacteroides* sp. population density, to the level of 7.20 and 6.54 log_10_CFU/g, respectively, at the age of 165 days (Table S1).

Moreover, a negative correlation was observed between synbiotics and potentially pathogenic microorganisms (*Clostridium* sp., *Enterococcus* sp., *Enterobacteriaceae* family, *E. coli*) as well as yeast population density (Figure 3). Synbiotics used as feed additives reduced *Clostridium* sp., *Enterococcus* sp., *Enterobacteriaceae* family, and *E. coli* counts to an average of 4.41, 5.42, 5.26, and 4.92 log_10_CFU/g, respectively (Table S1). Yeast population, which was the least abounded, presented by horizontal dendrograms in Figure 2, was substantially diminished from 4.00–4.10 log_10_CFU/g (average of 4.06 log_10_CFU/g) to 2.96–3.31 log_10_CFU/g (Table S1). Probiotics also considerably reduced the number of *Clostridium* sp., *Enterobacteriaceae* family, and *E. coli* in pigs’ feces, though only to the average of 5.81, 6.27, and 5.25 log_10_CFU/g, respectively (Table S1). However, these preparations did not have an impact on the amount of yeast in the piglets’ feces, which was similar to the samples obtained from the group of animals fed unmodified fodder.

Furthermore, the administration of unmodified feed to pigs resulted in a significant reduction of *Bifidobacterium* sp. and *Bacteroides* spp., as well as an increase of *Enterobacteriaceae* and *E. coli* abundance, especially after piglets have been weaned off breastfeeding by sows (29th day of life; Table S1).

### 3.2. Short-Chain Fatty Acids Fecal Concentrations

**Figure 4:**
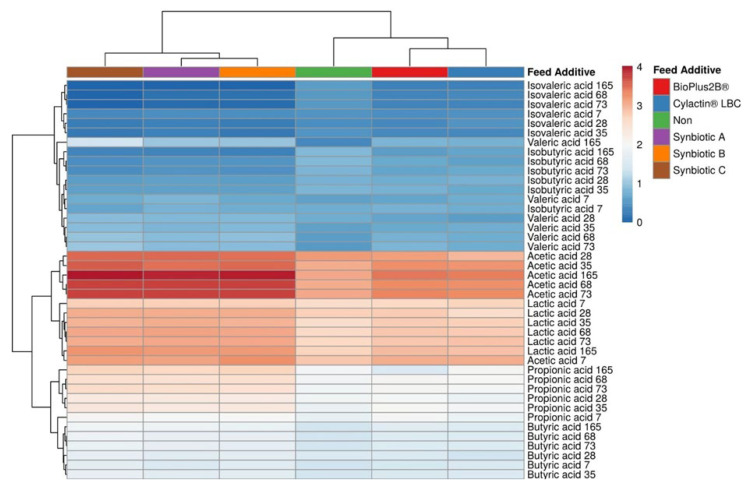
Heatmap presents changes in lactic acid, SCFAs, and BCFAs concentrations in piglets’ feces in correlation with the administrated feed additives. Columns correspond to the preparations used to modify piglets feed (directly or indirectly), whereas rows are assigned to analyzed organic acid and the day of piglets’ life when fecal samples were collected, namely, 7th and 28th (suckling piglets), 35th and 68th (weaned piglets), and 73rd and 165th (finisher pigs).

Results showed that piglets which were at first breastfed by sows whose fodder was supplemented with synbiotic preparations and then were fed directly with these diets exhibited a significant increase in the lactic acid and SCFAs, and a reduction of BCFA concentrations in feces. Both commercial probiotics, namely, BioPlus 2B^®^ (Chr. Hansen A/S, Horsholm, Denmark) and Cylactin^®^ LBC (DSM Nutritional Products Ltd., Kaiseraugst, Switzerland), substantially increased concentrations of only acetic acid (Figure 4). Additionally, BioPlus 2B^®^ (Chr. Hansen A/S, Horsholm, Denmark) considerably enhanced the rise of concentrations of lactic and butyric acids by about 4 µmol/g and 1 µmol/g, respectively, during the duration of the experiment, whereas Cylactin^®^ LBC (DSM Nutritional Products Ltd., Kaiseraugst, Switzerland) contributed to a significant increase of propionic acid by 1.93 µmol/g (Table S2). Nevertheless, vertical dendrograms in Figure 4 shows that the organic acids concentrations profile in fecal samples of pigs fed fodder with probiotics differ slightly from one observed in feces of piglets fed unmodified feed, on the contrary to feces of animals whose feed was supplemented with synbiotics.

**Figure 5:**
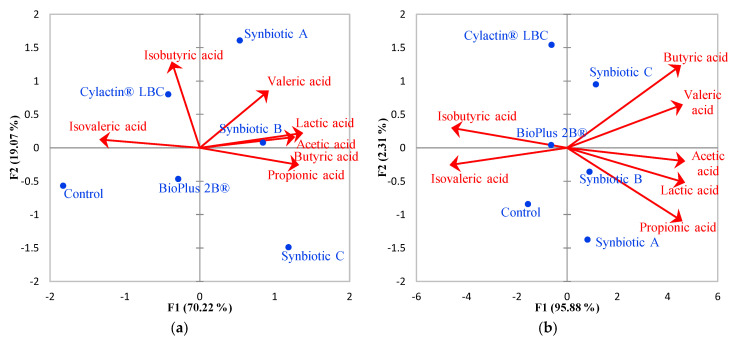

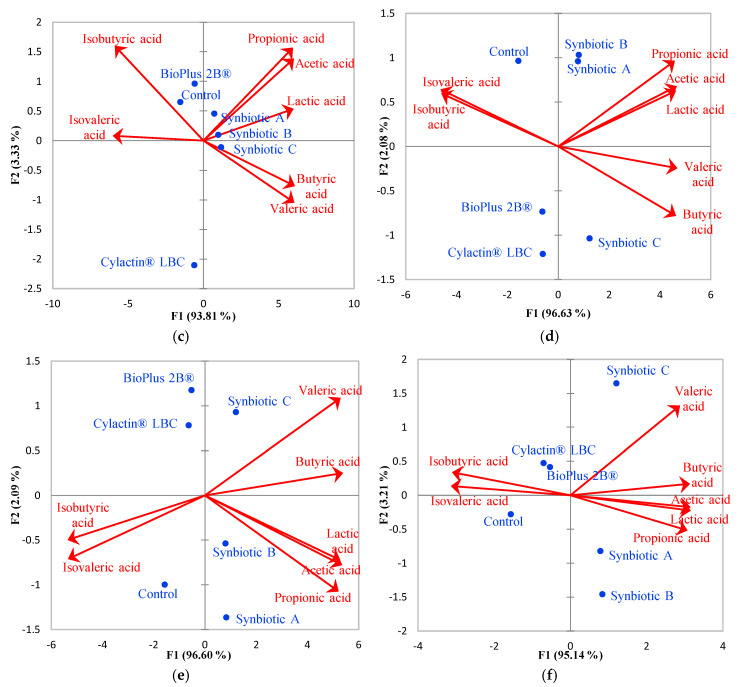
Correlation biplots display the PCA results of different preparations or its absence (blue dots), and their influence on lactate, SCFAs, and BCFAs synthesis and their concentrations (red vectors which represent variables) in pigs’ feces in different breeding stage: (**a**) 7th and (**b**) 28th day of life—piglets breastfed by sows; (**c**) 35th, (**d**) 68th, (**e**) 73rd, (**f**) and 165th day of life—feed additives administration to weaned piglets.

A strong positive correlation between synbiotics and lactic acid, as well as SCFA, concentrations in pigs’ fecal samples was demonstrated by the PCA (Figure 5). Furthermore, the negative correlation of these preparations with BCFAs concentrations in pigs’ feces was noted. During the conducted research, these correlations became more significant, which is shown in Figure 5a–f. Synbiotics contributed to a substantial increase of lactic acid concentrations in piglets’ feces by almost 10 µmol/g between the age of 7 and 165 days, whereas acetate concentrations were between 17.38 and 26.26 µmol/g (mean 24.11 µmol/g) on the 7th day of piglets’ life and after 165 days reached a level of 51.14–55.82 (mean 53.02 µmol/g; Table S2). Propionic acid concentrations in piglets’ feces were also increased, from an average of 7.64 µmol/g to 14.92 µmol/g when synbiotics were administrated with feed (Table S2). Among the analyzed preparations, only synbiotic A had a positive influence on butyric acid concentration in piglets’ feces, which was significantly increased from 4.70 to 6.55 µmol/g; however, this preparation did not have any effect on valeric acid concentration, which considerably rose when synbiotic B and C were used as feed additives, from the average of 2.04 µmol/g to 2.67 and 3.88 µmol/g, respectively (Table S2).

Based on the data collected, it was determined that only synbiotics administrated to sows which breastfed piglets or directly to weaned pigs substantially decreased concentrations of branch-chained fatty acids, namely, isobutyric and isovaleric acids, by more than 0.5 µmol/g. Nevertheless, horizontal dendrograms show that the concentrations of BCFAs along with valeric acid are considerably lower than the amount of lactic acid and the rest of SCFAs in pigs’ feces (Figure 4).

## 4. Discussion

Fecal microbiota composition is used to determine the status of microbial colonization in the GIT [32]. Interactions between the GIT microbiota, diet, and the mucosa is key to the gut health in pigs; therefore, feed additives that can influence the GIT health and functions are being looked for. They are evaluated for their ability to reduce the number of pathogenic bacteria, with a substantial increase in beneficial microbes in the GIT as well as stimulation of digestion [33]. Because of the insufficient amount of research on the matter of the synbiotics influence on pigs’ microbiota, most of the obtained outcomes were compared to studies conducted with the usage of probiotics.

The composition of the GIT microbiota is of vital importance, especially, an increased population of bacteria from *Lactobacillus* and *Bifidobacterium* along with *Bacteroides* genus might contribute to improvement in GIT health [34,35]. The more significant beneficial impact of newly developed synbiotics on the numbers of these bacteria genera was observed in our research than when probiotic preparations were used as piglets’ feed supplements. Mair at el. drew similar conclusions and additionally observed that inulin used separately did not impact the number of lactobacilli and *Bifidobacterium* sp. in digesta of weaned pigs as substantially as analyzed synbiotic containing this prebiotic along with *E. faecium*, *Lb. salivarius*, *Lb. reuteri*, and *B. thermophilum* [36]. Furthermore, a comparison of the gathered results with observations made by other researcher teams supports the hypothesis that probiotics have an impact on the number of beneficial bacteria, namely, *Lactobacillus* sp., *Bifidobacterium* sp., and *Bacteroides* sp. in fecal content of weaned pigs, although it is not as substantial as when the synbiotics are used. Among them, Choi et al. noted a slightly elevated number of *Bifidobacterium* sp. and *Lactobacillus* sp. when weaned pigs were fed fodder supplemented with multi-microbe probiotic described by Shim et al.; however, the number of these bacteria dropped between 14th and 28th day of the experiment, which was not in line with our results [37,38]. Additionally, Dowarah et al. showed a minor increase in *Bifidobacterium* sp. count in piglets’ feces when the feed was supplemented with probiotic strains, namely, *Lb. acidophilus* NCDC-15 or *Pediococcus acidilactici* FT28 [39]. Moreover, increased *Lactobacillus* sp. number in piglets’ fecal samples was also observed by Chiang et al. when *Lb. johnsonii* x-1d-2 and *Lb. mucosae* x-4w-1 were used as probiotics, whereas research conducted by Xu et al. showed that *S. cerevisiae* S288c strain contributed to the higher prevalence of *Bacteroides* sp. and *Lactobacillus* sp. in weaned piglets’ feces, which, despite being less substantial, was in accordance with our results [40].

Incorporating probiotics into pigs’ diet acted against pathogens as well, by competitive exclusion, influencing the immune system, or by the synthesis of antimicrobial substances; nevertheless, up until now, a substantial decrease in harmful bacteria had not been frequently reported [41]. Moreover, prebiotics, especially inulin, have been proven to have a positive effect on weaned piglets by modulation of the gut microbiota. They can promote the growth of beneficial microorganisms, and therefore, suppress the growth of pathogens [42]. The combination of pro- and prebiotics can act synergistically; hence, synbiotics were chosen for analysis of their impact on piglets GIT microbiota [43]. Among GIT microorganisms, some genera and species from *Enterobacteriaceae* bacterial family can be highly pathogenic and responsible for infections [44]. One of them is *E. coli*, which can be responsible for deaths or diarrhea in piglets, and as a consequence contribute to economic losses [16]. The results obtained by Grela et al. were similar to ours, and showed that synbiotics (*Lact. lactis* IBB500, *C. divergens* S1, *Lb. casei* 0915, *Lb. plantarum* 0862, *S. cerevisiae* 0141 + inulin) decreased the number of *Enterobacteriaceae* in pigs’ GIT, more substantially than when components of this preparation were used individually [45]. Chae et al. have also observed a lower prevalence of the *Enterobacteriaceae* family when synbiotics (*Enterococcus faecium* NCIMB 11181 + lactulose) were administrated to pigs, in comparison to the effect caused by pro- and prebiotics supplemented separately [46]. Our results also showed that synbiotic administration with feed had a more substantial effect on decreasing the abundance of *E. coli*, compared to probiotic use alone. Nevertheless, Zhao and Kim, as well as Nguyen et al., showed in their research that these beneficial microorganisms, used individually, can contribute to a decrease in the number of *E. coli* in pigs’ feces, which was in line with our results [47,48].

Another entero-pathogens that can contribute to the development of diarrhea in piglets are species belonging to *Clostridium* sp. and *Enterococcus* sp. [49]. It was observed that both probiotics and synbiotics added to sows’ feed resulted in lower *Clostridium* sp. and *Enterococcus* sp. counts in suckling piglets and decreased their number between 7th and 28th day of life. Choi et al. observed that the prevalence of *Clostridium* sp. in weaned pigs’ feces was lower when the multi-strain probiotic preparation was used; however, the number of these bacteria increased during the research [37]. On the contrary, our outcomes indicate that *Clostridium* sp. counts remained at a comparable level from weaning until the end of the study when probiotics were used, whereas synbiotics contributed to a further decrease in these bacteria counts. Analog dependency was observed for *Enterococcus* sp., which was similar to the results obtained by Li et al. [50].

Furthermore, it was observed that after piglets have been weaned off their mothers at the age of 29 days, the number of *Enterobacteriaceae* family representatives, including *E. coli*, as well as yeast population density, was increased considerably, regardless of the used feed additive or its absence. However, the abundance of *Enterobacteriaceae*, including *E. coli*, was substantially lower in piglets’ feces when fodder supplemented with synbiotics or probiotics was administrated to sows which fed suckling piglets, and then, after weaning, to piglets directly. Additionally, the administration of newly elaborated synbiotics helped to avoid the reduction of *Lactobacillus* sp. and *Bacteroides* sp. number as well as the increase of *Enterococcus* sp. number, as was observed in feces of piglets whose feed was unmodified or for whom probiotics were used, which only helped to maintain *Lactobacillus* sp. prevalence. The shift in the fecal microbiota of piglets after weaning was in line with the outcomes by Wei et al., who observed a higher number of *Enterococcus* sp. and *E. coli*, as well as a decreased population of *Lactobacillus* sp. in weaned pigs’ ileum [51].

Besides the microbial composition of fecal samples, concentrations of the organic acids (lactate, SCFAs, BCFAs) were changed when piglets were breastfed by sows whose feed was supplemented with new synbiotics or piglets who were fed, after weaning, feed modified with these preparations. SCFAs are well-known for their positive impact on GIT health and prevention from metabolic disorders [52]. Furthermore, these metabolites can improve breeding efficiency in swine and satisfy 5–28% of the caloric demands of animals, and along with lactate protect GIT from pathogens colonization through lowering pH levels [53,54,55]. On the other hand, BCFAs are products of amino acid breakdown by proteolytic bacteria, such as *Bacteroides* sp. or *Clostridium* sp. and these toxic metabolites might have a negative effect on piglets’ health [56,57].

Chaiyasut et al., who performed an analysis of the pro-, pre-, and synbiotic impact on SCFA production by rats’ intestinal microbiota, observed analog dependencies to ours [58]. In their research, synbiotic (*Lb. plantarum* HII11 + inulin) supplementation resulted in elevated concentrations of lactic acids and SCFAs (acetate, propionate, butyrate), contrary to the addition of probiotic strain, which did not affect organic acid production. Additionally, Martinez et al. presented in their work the positive effect of synbiotics on total SCFAs (acetate, propionate, butyrate) concentrations in the in vitro studies with the simulated proximal colon of pigs [52]. Similar results of synbiotic influence on SCFAs concentrations in pigs’ colon were obtained by Grela et al.; however, their studies also showed no effect of synbiotic on BCFAs concentrations in pigs’ distal colon, which was in opposition to our outcomes [45]. In comparison to the above-mentioned studies, newly developed synbiotics have a promising more beneficial effect on piglets’ microbiota metabolism, since they not only enhance the production of lactate and SCFAs but also suppress the synthesis of potentially harmful BCFAs.

The most considerable impact of synbiotic preparation on microbiota composition and the synthesis of organic acids was observed in the case of synbiotic C, which comprised the highest number of *Lactobacillus* sp. strains. Nevertheless, the performed study has its limitations. First of all, the sample size, despite being sufficient in order to perform statistical analysis and draw conclusions, was smaller than the whole herd. If all animals were studied, differences in results could be observed, although it would be time- and resource-consuming. Moreover, as was observed by Zommiti et al., the type of feed, as well as swine housing and other external factors, could impact the effectiveness of probiotics [59]. Therefore, the analyzed newly developed synbiotics should be studied further in the future keeping in mind these mentioned factors.

## 5. Conclusions

Outcomes gathered in this following research allowed us to conclude that both innovative synbiotic preparations and commercially available probiotics (BioPlus 2B^®^, Cylactin^®^ LBC) have a positive effect on piglets’ microbiota as well as organic acids production. However, the impact of probiotics used individually is less substantial than this of newly developed synbiotics. Moreover, probiotics do not contribute to the reduction of BCFAs concentrations that can negatively impact piglets’ health. In addition to that, the mixture of selected probiotic *Lactobacillus* spp. strains and *S. cerevisiae* ŁOCK 119 with inulin as a prebiotic compound prevent the negative effect of the weaning process on piglets microbiota which, on the contrary, was observed in the experimental group in which animals were administrated feed without any additives, as a significant increase in the prevalence of potentially pathogenic bacteria (*E. coli*), and a simultaneous reduction in the abundance of beneficial bacteria (*Lactobacillus* sp. and *Bifidobacterium* sp.).

In conclusion, the newly elaborated synbiotic preparations, by modulating GIT microbiota and its metabolism, can improve piglets’ health and prevent negative consequences of the weaning process in a more substantial way than commercially available probiotics.

## Figures and Tables

**Table 1:** Analyzed probiotics and synbiotics composition.

Feed Additive		Probiotic	Prebiotic
Synbiotics	Synbiotic A	*Lb. pentosus* ŁOCK 1094	Inulin
		*Lb. plantarum* ŁOCK 0860	
		*Lb. reuteri* ŁOCK 1092	
		*S. cerevisiae* ŁOCK 0119	
	Synbiotic B	*Lb. pentosus* ŁOCK 1094	
		*Lb. plantarum* ŁOCK 0860	
		*Lb. reuteri* ŁOCK 1092	
		*Lb. rhamnosus* ŁOCK 1087	
		*S. cerevisiae* ŁOCK 0119	
	Synbiotic C	*Lb. paracasei* ŁOCK 1091	
		*Lb. pentosus* ŁOCK 1094	
		*Lb. plantarum* ŁOCK 0860	
		*Lb. reuteri* ŁOCK 1092	
		*Lb. rhamnosus* ŁOCK 1087	
		*S. cerevisiae* ŁOCK 0119	
Probiotics	BioPlus 2B^®^	*Bacillus subtilis* CH201/DSM5749	- ^1^
		*Bacillus licheniformis* CH200/DSM574	
	Cylactin^®^ LBC	*Enterococcus faecium* NCIMB 10415	-

^1^ not applicable

**Table 2:** Feed components and chemical composition [30].

Ingredient [g/kg]	Sow	Pre-Starter (2–8 Weeks)	Starter (8–12 Weeks)	Grower (12–18 Weeks)	Finisher (12–24 Weeks)
Oats	100	-	-	-	150
Barley	250	220	370	125	200
Triticale	110	-	-	544	361
Wheat	362	300	400	125	150
Soybean meal	90	50	-	100	110
Soybean oil	10	20	20	15	5
Post-extraction soybean meal, heat-treated	40	25	140	-	-
Soybean, full-fat, heat-treated	-	50	-	-	-
Rapeseed meal	-	-	-	60	-
Whey permeate	-	50	-	-	-
LonoFish ^a^	-	50	25	-	-
Specilac ^b^	-	40	-	-	-
Lonacid Max ^c^	-	5	4	1	-
LonoGrain ^d^	-	150	-	-	-
Mycofix PLUS ^e^	-	-	1	-	-
Vitamin-mineral-amino acid premix ^1^	36	-	-	-	-
Vitamin-mineral-amino acid premix ^2^	-	40	-	-	-
Vitamin-mineral-amino acid premix ^3^	-	-	40	-	-
Vitamin-mineral-amino acid premix ^4^	-	-	-	30	25
	Chemical Composition				
Metabolizable energy (MJ/kg)	13.1	13.8	13.8	13.6	13.5
Crude protein (%)	16.5	18.8	17.9	16.8	15.6
Lysine (%)	0.88	1.56	1.28	1.05	0.95
Methionine + Cysteine (%)	0.59	0.82	0.76	0.66	0.57
Threonine (%)	0.58	0.87	0.79	0.67	0.56
Tryptophan (%)	0.20	0.30	0.20	0.19	0.18
Valine (%)	0.76	0.75	0.79	0.75	0.71
Calcium (%)	1.03	0.91	0.86	0.68	0.67
Phosphorus (%)	0.47	0.61	0.55	0.47	0.38
Vitamin A (IU/ kg)	12,500	14,000	20,500	12,000	7700
Vitamin D_3_ (IU/kg)	2000	2000	2000	2000	1540
Vitamin E (mg/kg)	80	84	100	63	100

^a^ protein source; ^b^ feed supplement rich in protein-lactose; ^c^ a dry mixture of phosphoric acid, formic acid, propionic acid, lactic acid, citric acid, acetic acid, and benzoic acid; ^d^ micronized wheat, barley, and maize; ^e^ toxin deactivator; ^1^ complementary feed (4%) L.K. T.CH. (Cargill Poland Sp. z o. o., Warsaw, Poland), Cargill Poland; ^2^ complementary feed (4%) PRESTART. T.CH. (Cargill Poland Sp. z o. o., Warsaw, Poland); ^3^ complementary feed (4%) START. T.CH. (Cargill Poland Sp. z o. o., Warsaw, Poland); ^4^ complementary feed (3/2.5%) GROW/FIN (Cargill Poland Sp. z. o. o, Warsaw, Poland).

**Table 3:** Cultivation condition of analyzed microorganisms.

Microorganisms	Polish Standard	Cultivation Conditions
Total no of anaerobic bacteria	PN-EN 4833-1:2013-12	37 °C, 48 h (anaerobically ^1^)
*Bifidobacterium* sp.	PN-EN 15785:2009E	
*Clostridium* sp.	PN-ISO 15213:2005	
*Bacteroides* sp.	- ^2^	37 °C, 48 h
*Lactobacillus* sp.	PN-EN 15787:2009	
*Enterococcus* sp.	PN-EN 15488:2009	
*Enterobacteriaceae* family	PN-ISO 21528-2:2005	
*E. coli*	PN-ISO 4832:2007	44 °C, 48 h
Yeast	PN-EN 15789:2009 (additional microscopic analysis)	30 °C, 72 h

^1^ To obtain the anaerobic condition anaerostats (AnaeroJar™; Oxoid™, Thermo Fisher Scientific, Waltham, MA, USA) along with anaerobic gas generating sachets (AnaeroGen™; Oxoid™, Thermo Fisher Scientific, Waltham, MA, USA) were used. ^2^ not applicable.

**Table 4:** Parameters applied in HPLC analysis of organic acid concentrations.

Parameter	Value/Type
Mobile Phase	0.005M H_2_SO_4_
Detector	refractive index (RI); ultraviolet (UV)
UV detector excitation wavelength (nm)	210
Autosampler	the loop dispensing valve with a syringe
Sample volume (μL)	10
Temperature (°C)	60
Flow rate (μL/min)	0.6
Duration (min)	35

## Supplementary Materials

The following are available online at https://www.mdpi.com/2076-2615/10/11/1999/s1. Table S1: Changes in the fecal microbiota of piglets at a different stage of rearing (suckling piglets (P), weaned piglets (W), and finisher pigs (F)) in regard to the administration of feed additives. Table S2: The mean concentration of lactic acid, SCFAs, and BCFAs in feces of piglets at a different stage of rearing (suckling piglets (P), weaned piglets (W), and finisher pigs (F)) in regard to the administration of feed additives.
