# Supplementary material for: The Effect of Recently Developed Synbiotic Preparations on Dominant Fecal Microbiota and Organic Acids Concentrations in Feces of Piglets from Nursing to Fattening
Source: Animals (Basel). 2020 Oct 30;10(11):1999. doi: 10.3390/ani10111999 (PMC7693995; doi:10.3390/ani10111999)
Supplement: Supplementary file 1 [file animals-10-01999-s001.zip › animals-970816-sup/Table S1.docx]

**Table S1.** Changes in the fecal microbiota of piglets at a different stage of rearing (suckling piglets (P), weaned piglets (W), and finisher pigs (F)) in regard to the administration of feed additives.

|  | **Sample collection (day)** | **Preparations administrated with feed** | | | | | |
| --- | --- | --- | --- | --- | --- | --- | --- |
|  |  | **Synbiotic A** | **Synbiotic B** | **Synbiotic C** | **BioPlus2B®** | **Cylactin® LBC** | **Non (Control group)** |
|  |  | **Log_10_ CFU/g (average ± SD)*** | | | | | |
|  | **The total number of anaerobic bacteria** | | | | | | |
| P | 7 | 9.4±0.14 ^C^ | 9.30±0.24 ^C^ | 9.29±0.10 ^C^ | 9.32±0.08 ^D^ | 9.20±0.22 ^B^ | 9.24±0.21 ^C^ |
|  | 28 | 9.03±0.08 ^B,C^ | 8.99±0.18 ^B, C^ | 8.85±0.26 ^B,C^ | 9.09±0.14 ^C,D^ | 9.02±0.17 ^B^ | 9.20±0.04 ^B,C^ |
| W | 35 | 8.86±0.28 ^b; B^ | 8.84±0.21 ^b; B,C^ | 8.92±0.13 ^b; B,C^ | 8.78±0.18 ^a,b; B,C^ | 8.24±0.34 ^a; A^ | 8.86±0.12 ^b; A,B,C^ |
|  | 68 | 8.78±0.25 ^b; B^ | 8.77±0.10 ^b; B,C^ | 8.69±0.30 ^b; B^ | 8.82±0.03 ^b; B,C^ | 8.12±0.02 ^a; A^ | 8.72±0.34 ^b; A,B^ |
| F | 73 | 8.65±0.07 ^B^ | 8.60±0.43 ^A,B^ | 8.54±0.09 ^A,B^ | 8.63±0.21 ^B^ | 8.49±0.29 ^A^ | 8.50±0.28 ^A^ |
|  | 165 | 8.14±0.25 ^A^ | 8.10±0.27 ^A^ | 8.10±0.22 ^A^ | 8.18±0.19 ^A^ | 8.16±0.18 ^A^ | 8.57±0.38 ^A^ |
|  | ***Bifidobacterium* sp.** | | | | | | |
| P | 7 | 6.84±0.21 ^A^ | 6.84±0.12 ^A^ | 6.83±0.22 ^A^ | 6.87±0.15 ^A^ | 6.83±0.26 ^A^ | 6.78±0.18 ^C^ |
|  | 28 | 7.51±0.33 ^b; B^ | 7.56±0.25 ^b; B^ | 7.71±0.17 ^b; B^ | 7.30±0.08 ^a,b; B^ | 7.30±0.22 ^a,b; B^ | 6.81±0.05 ^a; C^ |
| W | 35 | 7.43±0.21 ^b,c; B^ | 7.46±0.14 ^b,c; B^ | 7.52±0.13 ^c; B,C^ | 7.22±0.14 ^b,c; A,B^ | 7.10±0.02 ^b; A,B^ | 6.18±0.17 ^a; B^ |
|  | 68 | 7.55±0.01 ^c,d ;B^ | 7.61±0.04 ^c,d; B^ | 7.76±0.03 ^d; B,C^ | 7.46±0.16 ^c; B^ | 7.22±0.20 ^b; A,B^ | 5.81±0.13 ^a; A,B^ |
| F | 73 | 7.51±0.16 ^b; B^ | 7.56±0.21 ^b; B^ | 7.64±0.16 ^b; B,C^ | 7.37±0.32 ^b; B^ | 7.29±0.16 ^b; B^ | 5.72±0.25 ^a; A,B^ |
|  | 165 | 7.78±0.09 ^c; B^ | 7.83±0.11 ^c; B^ | 7.96±0.03 ^c; C^ | 7.39±0.22 ^b; B^ | 7.32±0.20 ^b; B^ | 5.48±0.19 ^a; A^ |
|  | ***Lactobacillus* sp.** | | | | | | |
| P | 7 | 6.40±0.17 ^A^ | 6.31±0.22 ^A^ | 6.40±0.11 ^A^ | 6.21±0.34 ^A^ | 6.37±0.30 ^A^ | 6.41±0.17 |
|  | 28 | 7.94±0.18 ^b; B^ | 8.13±0.12 ^b; B^ | 8.17±0.11 ^b; B^ | 6.55±0.46 ^a; A,B^ | 6.54±0.34 ^a; A,B^ | 6.39±0.12 ^a^ |
| W | 35 | 8.17±0.31 ^b; B,C^ | 8.25±0.22 ^b; B,C^ | 8.37±0.36 ^b; B,C^ | 6.96±0.12 ^a; B,C^ | 6.87±0.04 ^a; B,C^ | 6.03±0.16 ^a^ |
|  | 68 | 8.54±0.34 ^c; B,C,D^ | 8.65±0.07 ^c; B,C,D^ | 8.81±0.27 ^c; C,D^ | 7.23±0.04 ^b; B,C^ | 7.11±0.05 ^a,b; C,D^ | 6.24±0.34 ^a^ |
| F | 73 | 8.61±0.49 ^c; C,D^ | 8.69±0.30 ^c; C,D^ | 8.94±0.14 ^c; D^ | 7.32±0.16 ^b; C^ | 7.28±0.03 ^b; C,D^ | 6.22±0.37 ^a^ |
|  | 165 | 8.97±0.10 ^c; D^ | 9.03±0.35 ^c; D^ | 9.26±0.21 ^c; D^ | 7.42±0.24 ^b; C^ | 7.40±0.13 ^b; D^ | 6.16±0.27 ^a^ |
|  | ***Clostridium* sp.** | | | | | | |
| P | 7 | 7.10±0.43 ^E^ | 6.95±0.59 ^D^ | 6.93±0.40 ^D^ | 6.94±0.32 ^D^ | 7.10±0.13 ^E^ | 6.96±0.30 |
|  | 28 | 6.58±0.24 ^a,b; D,E^ | 6.56±0.06 ^a,b ;D^ | 6.31±0.23 ^a; C,D^ | 6.79±0.20 ^a,b; C,D^ | 6.85±0.21 ^b; D,E^ | 6.83±0.18 ^b^ |
| W | 35 | 6.24±0.25 ^a; C,D^ | 6.20±0.30 ^a; C,D^ | 6.12±0.13 ^a; C^ | 6.42±0.07 ^a; B,C^ | 6.53±0.15 ^a; C,D^ | 7.05±0.14 ^b^ |
|  | 68 | 5.59±0.14 ^a; B,C^ | 5.32±0.35 ^a; B,C^ | 5.27±0.06 ^a; B^ | 6.02±0.05 ^b; A,B^ | 6.22±0.12 ^b; B,C^ | 6.95±0.24 ^c^ |
| F | 73 | 5.30±0.36 ^a; B^ | 5.18±0.26 ^a; A,B^ | 5.15±0.15 ^a; B^ | 5.99±0.07 ^b; A,B^ | 6.06±0.24 ^b; A,B^ | 6.94±0.14 ^c^ |
|  | 165 | 4.54±0.34 ^a; A^ | 4.39±0.55 ^a, A^ | 4.30±0.43 ^a, A^ | 5.82±0.15 ^b; A^ | 5.79±0.17 ^b; A^ | 6.90±0.19 ^c^ |
|  | ***Enterococcus* sp.** | | | | | | |
| P | 7 | 6.66±0.32 ^B^ | 6.64±0.28 ^C^ | 6.71±0.30 ^C^ | 6.67±0.35 | 6.74±0.24 | 6.71±0.24 |
|  | 28 | 6.66±0.22 ^B^ | 6.57±0.11 ^C^ | 6.51±0.25 ^B,C^ | 6.66±0.22 | 6.77±0.10 | 6.78±0.25 |
| W | 35 | 6.60±0.17 ^B^ | 6.50±0.35 ^B,C^ | 6.45±0.23 ^B,C^ | 6.69±0.09 | 6.90±0.08 | 6.69±0.30 |
|  | 68 | 6.21±0.10 ^a; B^ | 6.15±0.22 ^a; B,C^ | 6.11±0.26 ^a; B,C^ | 6.58±0.17 ^b^ | 6.96±0.17 ^c^ | 6.84±0.09 ^b,c^ |
| F | 73 | 6.15±0.21 ^a; B^ | 5.99±0.03 ^a; A,B^ | 5.93±0.06 ^a; A,B^ | 6.66±0.26 ^b^ | 7.01±0.23 ^b^ | 6.92±0.31 ^b^ |
|  | 165 | 5.48±0.40 ^a; A^ | 5.46±0.12 ^a; A^ | 5.31±0.44 ^a; A^ | 6.22±0.36 ^b^ | 6.92±0.11 ^c^ | 6.99±0.12 ^c^ |
|  | ***Enterobacteriaceae* family** | | | | | | |
| P | 7 | 6.54±0.28 ^B^ | 6.56±0.07 ^C^ | 6.52±0.45 ^B^ | 6.65±0.16 ^A,B,C^ | 6.59±0.11 ^B^ | 6.56±0.31 ^A^ |
|  | 28 | 6.20±0.20 ^a,b; B^ | 6.13±0.31 ^a,b; B,C^ | 6.00±0.30 ^a; A,B^ | 6.44±0.23 ^a,b; A,B^ | 6.48±0.17 ^a,b; A,B^ | 6.65±0.07 ^b; A,B^ |
| W | 35 | 6.49±0.43 ^a; B^ | 6.56±0.31 ^a; C^ | 6.52±0.46 ^a; B^ | 6.91±0.12 ^a,b; C^ | 6.95±0.24 ^a,b; C^ | 7.43±0.18 ^b; D^ |
|  | 68 | 6.18±0.06 ^a; B^ | 6.17±0.05 ^a; B,C^ | 5.90±0.44 ^a; A,B^ | 6.80±0.14 ^b; B,C^ | 6.63±0.21 ^b; B^ | 6.90±0.08 ^b; A,B,C^ |
| F | 73 | 5.97±0.04 ^a; B^ | 5.94±0.08 ^a; B^ | 5.87±0.60 ^a; A,B^ | 6.80±0.14 ^b; B,C^ | 6.66±0.26 ^b; B,C^ | 7.02±0.09 ^b; B,C,D^ |
|  | 165 | 5.25±0.24 ^a; A^ | 5.31±0.19 ^a; A^ | 5.21±0.52 ^a; A^ | 6.30±0.25 ^b; A^ | 6.24±0.09 ^b; A^ | 7.10±0.21 ^c; C,D^ |
|  | ***E.coli*** | | | | | | |
| P | 7 | 6.36±0.32 ^C^ | 6.26±0.45 ^C^ | 6.49±0.20 ^D^ | 6.64±0.30 ^C^ | 6.49±0.34 ^C^ | 6.46±0.34 ^A^ |
|  | 28 | 5.76±0.23 ^a; B^ | 5.61±0.23 ^a; B^ | 5.76±0.41 ^a; B,C^ | 6.33±0.41 ^a,b; B,C^ | 6.34±0.03 ^a,b; C^ | 6.54±0.09 ^b; A^ |
| W | 35 | 5.95±0.04 ^a; B,C^ | 5.98±0.05 ^a; B,C^ | 5.90±0.35 ^a; C,D^ | 6.03±0.17 ^a; B,C^ | 6.02±0.21 ^a; B,C^ | 7.21±0.09 ^b; B^ |
|  | 68 | 5.59±0.18 ^a,b; A,B^ | 5.47±0.03 ^a,b; A,B^ | 5.64±0.11 ^a; B^ | 5.64±0.10 ^a,b; A,B^ | 5.84±0.20 ^b; B^ | 6.59±0.16 ^c; A^ |
| F | 73 | 5.46±0.22 ^a; A,B^ | 5.41±0.22 ^a; A,B^ | 5.36±0.15 ^a; B^ | 5.53±0.08 ^a; A,B^ | 5.75±0.17 ^a; A,B^ | 6.60±0.50 ^b; A,B^ |
|  | 165 | 5.13±0.18 ^b; A^ | 4.96±0.17 ^a,b; A^ | 4.66±0.26 ^a; A^ | 5.16±0.17 ^b; A^ | 5.34±0.08 ^b; A^ | 6.39±0.30 ^c; A^ |
|  | ***Bacteroides* sp.** | | | | | | |
| P | 7 | 7.50±0.04 ^A^ | 7.45±0.11 ^A^ | 7.47±0.23 ^A^ | 7.51±0.17 ^B^ | 7.33±0.44 | 7.40±0.20 ^C^ |
|  | 28 | 7.51±0.21 ^A^ | 7.48±0.21 ^A^ | 7.50±0.18 ^A^ | 7.46±0.15 ^B^ | 7.31±0.38 | 7.20±0.23 ^B,C^ |
| W | 35 | 7.60±0.28 ^b,c; A,B^ | 7.51±0.11 ^a,b,c; A^ | 7.63±0.07 ^c; A,B^ | 7.18±0.21 ^a,b,c; A,B^ | 6.97±0.10 ^a,b^ | 6.94±0.59 ^a; A,B,C^ |
|  | 68 | 7.72±0.18 ^b; A,B^ | 7.67±0.25 ^b; A^ | 7.83±0.12 ^b; A,B^ | 6.89±0.16 ^a; A^ | 6.87±0.12 ^a^ | 6.67±0.04 ^a; A,B^ |
| F | 73 | 7.74±0.16 ^c; A,B^ | 7.79±0.08 ^c; A,B^ | 7.99±0.01 ^c; B^ | 7.04±0.06 ^b; A^ | 7.20±0.08 ^b^ | 6.50±0.28 ^a; A^ |
|  | 165 | 8.02±0.11 ^c; B^ | 8.11±0.20 ^c; B^ | 8.63±0.21 ^d; C^ | 7.20±0.12 ^b; A,B^ | 7.28±0.14 ^b^ | 6.54±0.09 ^a; A^ |
|  | **Yeast** | | | | | | |
| P | 7 | 4.00±0.00 ^B,C^ | 4.10±0.17 ^C^ | 4.10±0.18 ^C^ | 4.16±0.28 | 3.79±0.09 ^A,B^ | 4.20±0.17 |
|  | 28 | 3.86±0.03 ^a,b; B,C^ | 3.93±0.04 ^a,b; C^ | 3.95±0.07 ^a,b; B,C^ | 4.10±0.18 ^b^ | 3.78±0.11 ^a; A^ | 4.15±0.21 ^b^ |
| W | 35 | 4.33±0.35 ^C^ | 4.49±0.20 ^D^ | 4.49±0.20 ^C^ | 4.20±0.17 | 4.15±0.21 ^B,C^ | 4.24±0.34 |
|  | 68 | 3.78±0.18 ^a,b; A,B^ | 3.73±0.20 ^a,b; B,C^ | 3.40±0.46 ^a; A,B^ | 4.09±0.30 ^b,c^ | 4.09±0.21 ^b,c; A,B,C^ | 4.50±0.28 ^c^ |
| F | 73 | 3.56±0.25 ^a,b; A,B^ | 3.39±0.16 ^a; A,B^ | 3.35±0.16 ^a; A^ | 4.01±0.03 ^b,c^ | 4.27±0.05 ^c; C^ | 4.51±0.62 ^c^ |
|  | 165 | 3.31±0.33 ^a; A^ | 3.22±0.15 ^a; A^ | 2.96±0.38 ^a; A^ | 3.91±0.04 ^b^ | 3.98±0.09 ^b; A,B,C^ | 4.44±0.06 ^c^ |

* Mean values per analyzed group of microorganisms labeled by different lowercase letters (a, b, c, d) were significantly different (one-way ANOVA with post hoc Tukey’s test; *p* < 0.05) among used feed additives (or its absence) per day of samples collection (rows), whereas capital letters (A, B, C, D, E) were differentiating significantly varied (one-way ANOVA with post hoc Tukey’s test; *p* < 0.05) mean values within preparations among days of samples collection (columns).
