# Supplementary material for: The Effect of Recently Developed Synbiotic Preparations on Dominant Fecal Microbiota and Organic Acids Concentrations in Feces of Piglets from Nursing to Fattening
Source: Animals (Basel). 2020 Oct 30;10(11):1999. doi: 10.3390/ani10111999 (PMC7693995; doi:10.3390/ani10111999)
Supplement: Supplementary file 1 [file animals-10-01999-s001.zip › animals-970816-sup/Table S2.docx]

**Table S2.** The mean concentration of lactic acid, SCFAs, and BCFAs in feces of piglets at a different stage of rearing (suckling piglets (P)’, weaned piglets’ (W), and finisher pigs’ (F)) in regard to the administration of feed additives.

|  | **Sample collection**  **(day)** | | **Preparations administrated with feed** | | | | | |
| --- | --- | --- | --- | --- | --- | --- | --- | --- |
|  |  |  | **synbiotic A** | **synbiotic B** | **synbiotic C** | **BioPlus 2B^®^** | **Cylactin® LBC** | **Non (Control group)** |
|  |  |  | **The mean concentration of acid [µmol/g] ± SD*** | | | | | |
|  | **lactic acid** | | | | | | | |
|  | **P** | 7 | 15.84±0.21 ^c,d; A^ | 15.78±0.27 ^c,d; A^ | 16.03±3.29 ^d; A^ | 14.66±0.05 ^b; A^ | 14.90±1.58 ^b,c^ | 13.42±0.69 ^a^ |
|  |  | 28 | 20.69±1.23 ^b; A,B^ | 20.48±4.43 ^b; B,C^ | 20.66±3.66 ^b; A,B,C^ | 16.26±2.30 ^a; A,B^ | 15.64±1.64 ^a^ | 13.41±1.53 ^a^ |
|  | W | 35 | 20.77±3.69 ^c; A,B^ | 19.74±1.72 ^b,c; A,B^ | 19.82±2.37 ^b,c; A,B^ | 16.19±2.24 ^a,b,c; A,B^ | 15.75±4.39 ^a,b^ | 13.66±1.43 ^a^ |
|  |  | 68 | 22.55±5.18 ^b; B^ | 21.50±4.06 ^b; C^ | 21.99±5.89 ^b; B,C^ | 16.86±2.66 ^a,b; A,B^ | 16.59±2.78 ^a,b^ | 14.02±2.54 ^a^ |
|  | F | 73 | 21.78±5.36 ^b; B^ | 21.80±2.46 ^b; C^ | 21.21±1.11 ^b; B,C^ | 17.16±1.43 ^a; A,B^ | 17.57±1.54 ^a^ | 14.48±2.22 ^a^ |
|  |  | 165 | 24.47±5.16 ^b; B^ | 24.74±3.60 ^b; C^ | 25.56±2.00 ^b; C^ | 18.73±3.33 ^a; B^ | 17.77±1.71 ^a^ | 15.33±1.31 ^a^ |
| SCFAs | **acetic acid** | | | | | | | |
|  | P | 7 | 22.71±3.23 ^a,b; A^ | 26.26±0.70 ^b; A^ | 23.36±6.90 ^a,b; A^ | 20.97±3.03 ^a,b; A^ | 21.13±4.19 ^a,b; A^ | 17.38±6.66 ^a^ |
|  |  | 28 | 33.85±2.94 ^b,c; B^ | 32.21±6.27 ^b,c; A^ | 34.46±7.56 ^c; A,B^ | 23.98±4.06 ^a,b; A,B^ | 24.49±5.29 ^a,b; A,B^ | 19.09±3.30 ^a^ |
|  | W | 35 | 32.55±7.05 ^b,c; A,B^ | 33.96±5.74 ^b,c; A,B^ | 36.15±8.01 ^c; B^ | 26.05±3.46 ^a,b; A,B,C^ | 20.97±4.11 ^a; A^ | 19.04±1.98 ^a^ |
|  |  | 68 | 42.35±3.94 ^b; B,C^ | 43.82±7.70 ^b; B,C^ | 43.82±7.70 ^b; B^ | 27.16±4.39 ^a; A,B,C^ | 26.99±3.06 ^a; A,B^ | 21.01±3.61 ^a^ |
|  | F | 73 | 43.77±6.18 ^b; C^ | 43.25±6.80 ^b; B,C^ | 43.76±5.35 ^b; B^ | 28.51±4.02 ^a; B,C^ | 28.02±2.93 ^a; A,B^ | 21.44±2.29 ^a^ |
|  |  | 165 | 51.14±5.94 ^c; C^ | 52.10±4.88 ^c; C^ | 55.82±2.51 ^c; C^ | 30.88±3.83 ^b; C^ | 29.43±5.31 ^b; B^ | 21.61±2.87 ^a^ |
|  | **propionic acid** | | | | | | | |
|  | P | 7 | 7.41±0.57 ^b,c; A^ | 7.68±1.21 ^c,d; A^ | 7.84 ± 0.15 ^d; A^ | 7.24±0.67 ^b;^ | 6.12±1.44 ^a; A^ | 5.97±0.25 ^a^ |
|  |  | 28 | 10.46±0.90 ^b; B,C^ | 9.85±0.58 ^b; A.B^ | 10.07 ± 0.91 ^b; A,B^ | 7.39±1.37 ^a^ | 6.77±0.95 ^a; A^ | 6.14±1.40 ^a^ |
|  | W | 35 | 10.13±1.74 ^b,c; B^ | 10.68±2.08 ^c; B,C^ | 10.77 ± 1.29 ^c; B,C^ | 7.60±1.01 ^a,b^ | 6.36±1.20 ^a; A,B^ | 6.36±2.60 ^a^ |
|  |  | 68 | 12.14±1.70 ^b; A,B,C^ | 12.82±1.43 ^b; C,D^ | 13.01 ± 1.69 ^b; C,D^ | 7.84±0.50 ^a^ | 7.63±0.63 ^a; A,B^ | 6.82±0.85 ^a^ |
|  | F | 73 | 13.04±2.24 ^b; B,C^ | 12.01±2.17 ^b; B,C^ | 13.57 ± 1.93 ^b; D^ | 7.91±1.01 ^a^ | 7.20±0.84 ^a; A,B^ | 6.81±0.96 ^a^ |
|  |  | 165 | 14.39±2.51 ^b; C^ | 15.21±2.24 ^b; D^ | 15.15 ± 2.50 ^b; D^ | 8.11±0.40 ^a^ | 8.05±1.68 ^a; B^ | 6.98±1.00 ^a^ |
|  | **butyric acid** | | | | | | | |
|  | P | 7 | 4.70±0.01 ^a,b,c; A^ | 5.17±1.15 ^b,c^ | 5.57 ± 1.57 ^c^ | 4.30±0.26 ^a,b,c; A^ | 4.60±0.44 ^a,b^ | 3.78±0.69 ^a^ |
|  |  | 28 | 5.13±0.74 ^a,b; A,B^ | 5.30±0.83 ^a,b^ | 5.81 ± 1.56 ^b^ | 4.62±0.28 ^a,b; A,B^ | 4.70±0.99 ^a,b^ | 3.87±0.90 ^a^ |
|  | W | 35 | 5.14±0.73 ^a,b; A,B^ | 5.39±0.75 ^a,b^ | 5.70 ± 1.33 ^b^ | 4.56±0.55 ^a,b; A,B^ | 4.69±0.58 ^a,b^ | 3.98±0.84 ^a^ |
|  |  | 68 | 5.74±0.99 ^b,c; A,B^ | 5.72±0.92 ^b,c^ | 6.49±1.05 ^c^ | 4.81±0.63 ^a,b; A,B^ | 4.90±0.75 ^a,b^ | 3.98±0.51 ^a^ |
|  | F | 73 | 5.79±0.88 ^b; A,B^ | 5.78±1.10 ^b^ | 6.17±1.07 ^b^ | 4.83±0.51 ^a,b; A,B^ | 4.93±0.81 ^a,b^ | 4.14±0.63 ^a^ |
|  |  | 165 | 6.55±1.25 ^b; B^ | 6.31±0.92 ^b^ | 6.85±1.91 ^b^ | 5.40±0.80 ^a,b; B^ | 4.99±1.00 ^a,b^ | 3.99±0.92 ^a^ |
|  | **valeric acid** | | | | | | | |
|  | P | 7 | 2.26±0.46 | 2.00±0.09 ^A^ | 2.08±0.02 ^A^ | 1.92±0.33 | 2.07±0.12 | 1.83±0.13 |
|  |  | 28 | 2.34±0.02 | 2.36±0.64 ^A,B^ | 2.42±0.52 ^A^ | 1.96±0.30 | 2.13±0.38 | 1.80±0.72 |
|  | W | 35 | 2.28±0.36 | 2.34±0.17 ^A,B^ | 2.46±0.52 ^A^ | 2.02±0.35 | 2.12±0.34 | 1.88±0.42 |
|  |  | 68 | 2.47±0.23 ^a,b^ | 2.55±0.49 ^b; A,B^ | 2.64±0.47 ^b; A^ | 2.16±0.38 ^a,b^ | 2.13±0.21 ^a,b^ | 1.81±0.08 ^a^ |
|  | F | 73 | 2.50±0.49 ^b^ | 2.51±0.40 ^b; A,B^ | 2.75±0.69 ^b; A^ | 2.24±0.24 ^a,b^ | 2.13±0.23 ^a,b^ | 1.72±0.27 ^a^ |
|  |  | 165 | 2.78±0.16 ^b^ | 2.67±0.39 ^b; B^ | 3.88±0.65 ^c; B^ | 2.24±0.47 ^b^ | 2.20±0.41 ^b^ | 1.48±0.33 ^a^ |
| BCFAs | **isobutyric acid** | | | | | | | |
|  | P | 7 | 2.15±0.16 ^B^ | 2.09±0.45 ^B^ | 1.94±0.26 | 2.17±0.05 | 2.12±0.11 | 2.07±0.18 |
|  |  | 28 | 1.85±0.03 ^A,B^ | 1.81±0.37 ^A,B^ | 1.82±0.39 | 2.14±0.58 | 2.16±0.10 | 2.23±0.47 |
|  | W | 35 | 1.88±0.27 ^A,B^ | 1.85±0.31 ^A,B^ | 1.87±0.32 | 2.18±0.72 | 2.02±0.58 | 2.27±0.39 |
|  |  | 68 | 1.63±0.48 ^a; A,B^ | 1.67±0.36 ^a,b; A,B^ | 1.56±0.37 ^a^ | 2.00±0.49 ^a,b^ | 1.90±0.12 ^a,b^ | 2.33±0.38 ^b^ |
|  | F | 73 | 1.67±0.36 ^a,b; A,B^ | 1.61±0.28 ^a; A,B^ | 1.53±0.37 ^a^ | 1.91±0.26 ^a,b^ | 2.02±0.52 ^a,b^ | 2.28±0.24 ^b^ |
|  |  | 165 | 1.41±0.40 ^a,b; A^ | 1.34±0.30 ^a; A^ | 1.39±0.27 ^a,b^ | 1.83±0.21 ^a,b,c^ | 1.96±0.25 ^b,c^ | 2.37±0.53 ^c^ |
|  | **isovaleric acid** | | | | | | | |
|  | P | 7 | 1.53±0.06 ^a,b; D^ | 1.52±0.12 ^a; C^ | 1.49±0.14 ^a; B^ | 1.53±0.22 ^a,b^ | 1.57±0.09 ^a,b^ | 1.66±0.16 ^b^ |
|  |  | 28 | 1.35±0.21 ^a,b; C^ | 1.30±0.26 ^a; B,C^ | 1.29±0.21 ^a; B^ | 1.49±0.09 ^a,b^ | 1.51±0.11 ^a,b^ | 1.67±0.34 ^b^ |
|  | W | 35 | 1.35±0.16 ^a; C^ | 1.23±0.14 ^a; B^ | 1.26±0.28 ^a; B^ | 1.45±0.19 ^a,b^ | 1.49±0.15 ^a,b^ | 1.67±0.22 ^b^ |
|  |  | 68 | 1.16±0.21 ^b; B^ | 1.13±0.21 ^b; B^ | 0.89±0.10 ^a; A^ | 1.43±0.13 ^c^ | 1.47±0.17 ^c^ | 1.73±0.19 ^d^ |
|  | F | 73 | 1.13±0.09 ^a,b; B^ | 1.11±0.18 ^a,b; A,B^ | 0.91±0.10 ^a; A^ | 1.41±0.16 ^b,c^ | 1.42±0.28 ^b,c^ | 1.71±0.35 ^c^ |
|  |  | 165 | 0.89±0.10 ^a; A^ | 0.85±0.14 ^a; A^ | 0.81±0.13 ^a; A^ | 1.36±0.10 ^b^ | 1.34±0.10 ^b^ | 1.79±0.12 ^c^ |

* Mean values per analyzed group of microorganisms labeled by different lowercase letters (a, b, c, d) were significantly different (one-way ANOVA with post hoc Tukey’s test; *p* < 0.05) among used feed additives (or its absence) per day of samples collection (rows), whereas capital letters (A, B, C, D) were differentiating significantly varied (one-way ANOVA with post hoc Tukey’s test; *p* < 0.05) mean values within preparations among days of samples collection (columns).
